# Impacts of *Paraburkholderia phytofirmans* Strain PsJN on Tomato (*Lycopersicon esculentum* L.) Under High Temperature

**DOI:** 10.3389/fpls.2018.01397

**Published:** 2018-10-18

**Authors:** Alaa Issa, Qassim Esmaeel, Lisa Sanchez, Barbara Courteaux, Jean-Francois Guise, Yves Gibon, Patricia Ballias, Christophe Clément, Cédric Jacquard, Nathalie Vaillant-Gaveau, Essaïd Aït Barka

**Affiliations:** ^1^SFR Condorcet FR CNRS 3417, Unité de Recherche Résistance Induite et BioProtection des Plantes, UFR Sciences Exactes et Naturelles, Université de Reims Champagne-Ardenne, Reims, France; ^2^UMR 1332 Biologie du Fruit et Pathologie, INRA, Villenave-d’Ornon, France

**Keywords:** *Paraburkholderia phytofirmans* strain PsJN, high temperature, chlorophyll fluorescence, gas exchange, tomato (*Lycopersicon esculentum* L.)

## Abstract

Abnormal temperatures induce physiological and biochemical changes resulting in the loss of yield. The present study investigates the impact of the PsJN strain of *Paraburkholderia phytofirmans* on tomato (*Lycopersicon esculentum* Mill.) in response to heat stress (32°C). The results of this work showed that bacterial inoculation with *P. phytofirmans* strain PsJN increased tomato growth parameters such as chlorophyll content and gas exchange at both normal and high temperatures (25 and 32°C). At normal temperature (25°C), the rate of photosynthesis and the photosystem II activity increased with significant accumulations of sugars, total amino acids, proline, and malate in the bacterized tomato plants, demonstrating that the PsJN strain had a positive effect on plant growth. However, the amount of sucrose, total amino acids, proline, and malate were significantly affected in tomato leaves at 32°C compared to that at 25°C. Changes in photosynthesis and chlorophyll fluorescence showed that the bacterized tomato plants were well acclimated at 32°C. These results reinforce the current knowledge about the PsJN strain of *P. phytofirmans* and highlight in particular its ability to alleviate the harmful effects of high temperatures by stimulating the growth and tolerance of tomato plants.

## Introduction

The biggest challenge facing the world at present is climate change as it will affect the geographical distribution of vegetation types, ecosystem processes, primary production, and abundance of plant species (Malcolm et al., 2006; Lesk et al., 2016). High temperature is a main environmental factor that often limits the growth and productivity of important crop species and also lead to a great extent a series of morphological, biochemical, and physiological changes (Wahid et al., 2007; Barnabás et al., 2008). Rising temperature can cause a change in growth periods and crops, leading to a high risk of survival of specific species (Mendelsohn et al., 2016). High temperatures may benefit some crops but harm others owing to the increased evapotranspiration and thermal damage. In general, plants can develop different adaptation mechanisms to avoid heat stress (Bita and Gerats, 2013). To satisfy the demand for food, it is necessary to develop crops with high resistance to heat stress. Extreme temperature is one of the most severe and damaging environmental factors that affect the integrity of plant cells. The increase in temperature adversely affects the quantity and quality of all plant species, including tomatoes (Rodriguez-Ortega et al., 2016). The exposure of plants to long- or short-term high temperatures has a negative impact on the fruits by altering specific physiological processes in male reproductive development (Sato et al., 2000, 2006) and reducing crop production (Mohammed and Tarpley, 2011).

Photosynthesis is one of the key physiological heat-sensitive processes in plants affected by thermal stress and can be completely inhibited before any other symptom is detected (Berry and Bjorkman, 1980; Camejo et al., 2005). Photosynthesis is mainly affected by the reduction of foliar expansion, the inadequate functioning of the photosynthetic machinery, and leaf senescence (Wahid et al., 2007; Gururani et al., 2015a,b). High temperatures damage the process of photosynthesis by altering the photosynthetic pigments (Camejo et al., 2006), reducing the activity of the PSII (Camejo et al., 2005), and damaging the regeneration capacity of RuBP (Wise et al., 2004). Yamori et al. (2014) reported that the photosynthetic reactions in C3 plants showed a higher temperature homeostasis than photosynthesis under high growth temperatures. The same authors reported that C3 plants generally have a greater capacity of acclimatization of the temperature of photosynthesis in a wide range of temperatures: CAM plants acclimated the day and night photosynthetic process differently to temperature, and C4 plants were adapted to warmer environments. The metabolism of carbohydrates and other organic compounds can act as a source of energy. Assimilated carbon plays a crucial role in the survival of plants subjected to abiotic stress, such as high temperatures (Stobrawa and Lorenc-Plucinska, 2007).

The greater part of the energy of a plant is generated by photosynthesis. However, to grow, plants require significant amounts of nitrate, phosphate, and other minerals that are often not readily available in the soil. Numerous bacterial species associated with plants have exerted beneficial effects on growth promotion by providing many limiting compounds. PGPR can improve plant growth under conditions of abiotic stress (Avis et al., 2008; Bulgarelli et al., 2013). PGPR includes several genera of bacteria such as *Rhizobium, Bacillus, Pseudomonas*, and *Burkholderia*. In this context, the members of *Paraburkholderia* are among the most abundant bacteria with positive and impressive effects to improve the growth and fitness of plants (Compant et al., 2008). *P. phytofirmans* PsJN was isolated from onion roots infected with *Glomus vesiculiferum* (Frommel et al., 1991). As an endophyte, the PsJN strain of *P. phytofirmans* contributes to the fitness and development of the plants, presenting beneficial traits that can be exploited in agricultural biotechnology (Ait Barka et al., 2000, 2002, 2006; Compant et al., 2005b; Miotto-Vilanova et al., 2016; Su et al., 2016; Timmermann et al., 2017). The PsJN strain of *P. phytofirmans* is capable of colonizing the rhizosphere and several organs of several plant species (Compant et al., 2011), and induces resistance to biotic stress (Ait Barka et al., 2000, 2002; Pinedo et al., 2015; Miotto-Vilanova et al., 2016; Su et al., 2017). In addition, the PsJN strain of *P. phytofirmans* can improve the performance of plants under abiotic stress through a series of physiological and biochemical changes. Under stress due to drought, *P. phytofirmans* strain PsJN has significant effects on agronomic and physiological parameters such as shoot and root biomass, chlorophyll content, gas exchange, photochemical efficiency (Fv/Fm), and maize and wheat yield (Naveed et al., 2014a,b). In addition, in short-term and constant salt stress, Pinedo et al. (2015) have reported that *Arabidopsis thaliana* plants bacterized with the PsJN strain have significantly improved the number of siliques, fresh weight, plant height, and proline content. When potato plants (*Solanum tuberosum* L.) were inoculated with the PsJN strain, they showed a greater number of tubers and tuber weight under heat stress (Bensalim et al., 1998). In grapevine seedlings (*Vitis vinifera* L.), Ait Barka et al. (2006) found that the PsJN strain improved the photosynthetic activity and the level of photosynthesis, the growth of the plant, and the biomass at 4°C. The grapevine seedlings showed a balance of carbohydrates favorable to cold tolerance and also significantly improved the levels of starch, proline, and phenolics (Fernandez et al., 2012).

The consequences of abiotic stress on pigments, electron transport systems, photosystems, enzymatic activities related to photosynthesis, fluorescence of chlorophyll, and gas exchange in plants have been reported. However, these studied (Wise et al., 2004; Wahid et al., 2007; Abdelmageed and Gruda, 2009; Li et al., 2009; Sawicki et al., 2012, 2015). However, these studies have focused mainly on the adaptive responses of plants to stress, but less attention has been paid to the impact of beneficial bacteria on the ability of plants to recover under stress.

The specific objectives of the present investigation are to evaluate how the presence of *P. phytofirmans* strain PsJN impacts the physiology of tomato plants, particularly the responses related to photosynthesis, under conditions of moderately high temperature (32°C) compared to normal conditions (25°C). These objectives were achieved by measuring photosynthesis and parameters of carbohydrate metabolism such as gas exchange (Pn rate, g_w_, internal CO_2_ concentrations, and transpiration rate), chlorophyll fluorescence, and pigment content. In parallel, the variation of several biochemical parameters, including the main carbohydrates and the organic acids, were also controlled against thermal stress. The present study suggests that the PsJN strain of *P. phytofirmans* induces adaptive mechanisms to avoid negative impacts at high temperatures in tomato plants.

## Materials and Methods

### Bacterial Inoculum

The bacterial inoculum was cultured by transferring two loops of *P. phytofirmans* PsJN to 100 ml of King’s B liquid medium (King et al., 1954) and supplemented with 50 μg/l kanamycin incubated with shaking at 180 rpm at 28°C for 24 h. The bacteria were collected by centrifugation (4500 × g for 5 min at 4°C) and washed twice with phosphate buffered saline (PBS) (10 mM, pH 6.5). The pellet was resuspended in sterilized PBS, and the bacterial concentration was adjusted to 10^6^ CFU/ml with PBS (Ait Barka et al., 2006).

### Plant Growth Conditions and Bacterial Inoculation

Tomato seeds (Tumbling Tom Red cv.) were planted in a seedling tray of plantlets filled with unsterilized peat moss and covered with perlite. The roots of 23-day-old seedlings were immersed in the bacterial inoculum 10^6^ CFU/ml or PBS (control) for 2 min, and then transplanted into larger pots of 3 L volume (1.5 kg peat moss). The plants were transferred to the greenhouse in which the temperature was maintained at 32°C under 16 h of light and at 27°C for 8 h of darkness. The other set of plantlets (controls) was maintained under standard conditions (25°C/20°C, 16 h day/8 h night). The illumination was 750 μmol/m^2^s, and the relative humidity of the air remained at 70%. The plants were irrigated with the Hoagland nutrient solution (Adam, 2008). Each treatment was repeated in two independent experiments, each with three replicates of seven plants (*n* = 21).

### Epiphytic and Endophytic Colonization

The rhizoplane and the endophytic colonization of roots and shoots by the gfp-marked *P. phytofirmans* strain PsJN were determined on different days post-inoculation (0, 2, 7, 14, and 21 dpi). Each time, three samples (1 g each) of each treatment (without inoculum and inoculated with the PsJN strain) were randomly selected. For the epiphytes, the roots were retrieved from the soil and placed in a sterile tube containing 5 ml of sterile PBS and vortexed for 1 min at 240 rpm. To determine endophytic colonization, the roots, shoots, and leaves were disinfected on the surface first in 70% ethanol for 3 min, followed by commercial bleach at 1% containing 0.01% Tween 20 solution for 1 min and then they were rinsed three times with sterile distilled water. The samples were grounded and homogenized with 1 ml of sterile PBS. The homogenates were serially diluted, in microtiter plates, with sterile H_2_O in 10-fold dilutions and 10 μL aliquots were plated in King’s solid B medium supplemented with 50 mg/ml kanamycin and cycloheximide (50 μg/ml). The plates were incubated at 28°C for 72 h, and the CFU number was determined by counting the bacterial colonies under a fluorescence stereomicroscope (model MZ FLIII, Leica, Heerbrugg, Switzerland) equipped with a GFP 1 filter (Leica, Switzerland).

### Leaf Gas Exchange

The portable photosynthesis system was measured using the instrument (LI-6400 XT; Li-Cor, Lincoln, NE, United States) using equations developed by (Von Caemmerer and Farquhar, 1981). The system is equipped with a 6400-40 leaf chamber fluorometer. The temperature and humidity of the air were maintained at 25 or 32°C and 60%, respectively. The actinic light provided by a red and blue light-emitting diode was set at 750 μmol m^-2^ s^-1^ and the CO_2_ concentration at a constant level of 400 ppm using a CO_2_ injector with a liquid CO_2_ cartridge source at high pressure. The gas exchange measurements were made using the fifth leaf of the apex of the plant for three plants replicated by the condition.

### Chlorophyll Fluorescence

The fluorescence of chlorophyll reflects the functionality of the photosynthetic apparatus because it is the result of absorbed light. Chlorophyll fluorescence was measured directly for 45 days in tomato leaves after 10 days post-inoculation. The fluorescence of foliar chlorophyll was quantified by controlling the quantum Y (II) photochemical yields using MONITORING-PAM (Walz, Effeltrich, Germany). The fluorometer uses a repetitive saturation pulse method and provides an automatic regimen of data collection as described by Su et al. (2016). Pulses of light (1 s, 3500 μmol m^-2^ s^-1^) were applied every 20 min. The saturation pulse detected and calculated automatically the fluorescence parameters of leaves. The measurements were recorded with WinControl-3 software (Heinz Walz GmbH, Inc., Effeltrich, Germany).

### Photosynthetic Pigment Content

Samples of fresh leaves (100 mg) of each treatment (in triplicate) were crushed with N_2_ and added to 5 ml of 80% acetone (v/v) adjusted with CaCO_3_ 0.5% (w/v) to avoid the acidification of chlorophyll. The extract was centrifuged at 3000 × *g* for 15 min at 4°C, and the supernatant was used to measure the pigment concentrations by using a spectrophotometer according to (Wellburn, 1994). The absorbance of the extracted pigments was read at λ = 663, 645, and 470 nm using a UV-VIS spectrophotometer (SmartSpec 3000, United States). The pigment concentrations (mg/l) of chlorophyll a (Ch.a), b (Ch.b), total chlorophyll (Ch.a + Ch.b), and carotenoids were calculated using the following equations:

$$\begin{array}{c} \mathrm{Ch}.a=\left(12.25\times A663\right)-\left(2.79\times A645\right)\\ \mathrm{Ch}.b=\left(21.5\times A645\right)-\left(5.1\times A663\right)\\ \mathrm{Ch}.a+\mathrm{Ch}.b=7.15\times A663+18.71\times A645\\ \mathrm{Carotenoids}=\left(1000\times A470-1.82\;\mathrm{Ch}.a-85.02\;\mathrm{Ch}.b\right)/198 \end{array}$$

Results were displayed in milligrams per gram of fresh weight (mg/g FW).

### Organic Compounds

Using 20 mg of fresh samples, the organic compounds were extracted twice in the presence of 80% ethanol (v/v) and once with 50% ethanol (v/v) for the next analysis.

First, the supernatant was prepared in 96-well microplates using the Starlet (Hamilton) pipetting robots. The sugars (sucrose, fructose, and glucose) were determined according to the method of (Jelitto et al., 1992), malate (Nunes-Nesi et al., 2007), total amino acids (Bantan-Polak et al., 2001), and proline (Troll and Lindsley, 1955). Protein and starch were analyzed in pellets according to (Bradford, 1976) and (Hendriks et al., 2003), respectively. Absorption was measured at 340 and 570 nm using an MP96 microplate reader (SAFAS) as described by (Biais et al., 2014).

### RNA Extraction, cDNA Synthesis, and Real-Time PCR

For gene expression, bacterized or non-bacterized tomato leaves grown at 25 or 32°C were harvested 7, 14, and 21 days after inoculation and triturated in liquid nitrogen. Total RNAs of 100 mg were extracted from each sample using Extract-All reagent (Eurobio, France). An amount of 1 μg of RNA was used for reverse transcription using the Verso cDNA synthesis kit (Thermo Fisher Scientific) according to the manufacturer’s instructions. The transcription levels were determined by qPCR using the CFX 96TM real-time system (Bio-Rad, France) and the combined SYBR Green Master PCR kit as recommended by the manufacturer (Applied Biosystems). The PCR conditions were used as described by Miotto-Vilanova et al. (2016). Briefly, PCR reactions were carried out, in duplicate, in 96-well plates (15 μl per well) in a buffer containing 1× SYBR Green I mix (including Taq polymerase, dNTPs, and SYBR Green dye), forward and reverse primers 280 nM, and 1:10 dilution of reverse transcription RNA. After denaturation at 95°C for 15 min, the amplification occurred in a two-step procedure: 15 s of denaturation at 95°C and 1 min of annealing/extension at 60°C, for a total of 30 cycles. Identical thermal cycling conditions were used for all objectives. The specific primers were designed in this study using the First BLAST software^1^ and are listed as (**Supplementary Table S1**). The results correspond to mean ± standard deviation (SD) of two independent experiments, each performed in duplicate. The relative expression of the gene was determined with the double induction formula: 2^-ΔΔCt^, where ΔΔCt = (Ct GI [unknown sample] – Ct GI [reference sample]) – (Ct reference genes [unknown sample] – Ct genes from reference [reference sample]). GI is the gene of interest. *EF1a* and *ACTIN* are used as internal controls. The reference sample is the “control 25°C” sample, chosen to represent 1× expression of the gene of interest.

### Statistical Analysis

The results of the statistical analysis of this study were performed using the GraphPad Prism 5.0 software (GraphPad Software Inc., La Jolla, CA, United States). The data were made using a two-way analysis of variance (ANOVA), while the chlorophyll fluorescence data were statistically analyzed using the Student’s *t*-test using Microsoft Excel. The significance was established at *P* < 0.05.

## Results

The rhizoplane of tomato roots was colonized by *P. phytofirmans* strain PsJN cells immediately after the inoculation of the soil, reaching 6.85 ± 04 log10 CFU/g FW (fresh weight) of the root tissue. The PsJN::*gfp*2x population peaked at 24 h post-inoculation and then decreased slowly until reaching 4.8 ± 0.39 log10 CFU/g FW 21 dpi (**Supplementary Table S2**). However, PsJN::*gfp*2x cells were not detected in the roots until 7 dpi, when the endophytic population PsJN::*gfp*2x reached 4.05 ± 0.19 log10 CFU/g FW and then slowly decreased to reach 2.96 ± 0.39 log10 CFU/g FW 21 dpi (**Supplementary Table S2**).

The endophytic presence of the PsJN::*gfp*2x cell in the stem was detected only on the 7th dpi when the PsJN::*gfp*2x population reached 3.31 ± 0.40 log10 CFU/g FW, and then slowly decreased to reach 1.61 ± 0.56 log10 CFU/g FW 21 dpi (**Supplementary Table S2**). However, no PsJN::*gfp*2x cells were detected in the tissues of the leaves throughout the experiment. The pattern of the colonization process was not affected by the heat treatment.

In the present study, the presence of *P. phytofirmans* PsJN caused a significant promotion of the aerial part of tomato in comparison with the control plants. All bacterized seedlings survived rhizosphere bacterization and performed better than non-bacterized seedlings. However, the temperature has no impact on PsJN performance since no significant difference was observed between plants growing at 25°C and those subjected to 32°C (**Supplementary Figure S1**).

### Stimulation of Gas Exchange After Plant Bacterization in Response to a Temperature Increase

**FIGURE 1:**
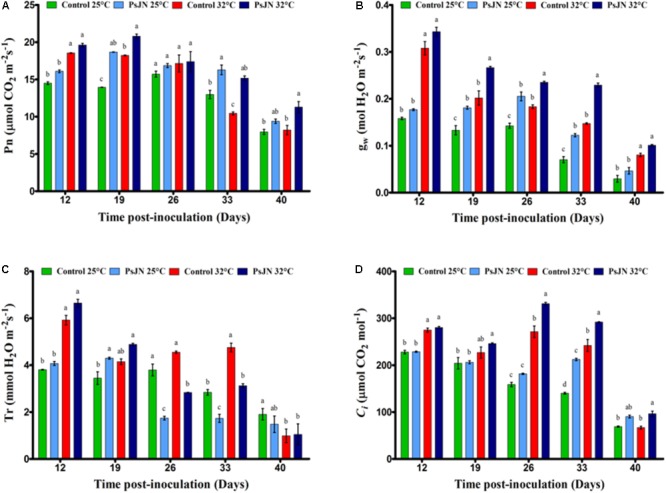
The net photosynthetic rate, Pn **(A)**; stomatal conductance, gs **(B)**; transpiration rate, Tr **(C)**; and internal CO_2_ concentrations, *Ci* **(D)** in leaves of mock-treated and bacterized tomato plants. Measurements were conducted at both conditions 25 and 32°C. Same letters indicate non-significant differences among all conditions. Data (mean ± SE) are averages of three independent experimental replicates, each with three plants per treatment (*n* = 9).

In the early stages of tomato development (control), Pn was approximately 15 mmol/m^2^ and then decreased through stages of development (**Figure 1A**). Our results indicate that Pn increased with high temperatures until day 26 and then began to decrease. In addition, the highest level of Pn had been reached in bacterized tomato plants compared to non-bacterized plants, whatever the temperature applied, except on day 26 after inoculation.

Similar to Pn, the g_w_ was significantly higher in the early stages of development (control) and decreased with the age of the plant (**Figure 1B**). The value of g_w_ of the non-inoculated plants increased because of the high-temperature treatment. There was a significant increase in the body weight of plants inoculated with PsJN compared to non-inoculated plants, regardless of temperature, except 12 and 40 dpi.

The transpiration rate (Tr) of the control plants showed a significant decrease during the stages of flowering and fruit set in both temperature conditions (**Figure 1C**). Tr increased with higher temperature after 12 and 33 dpi and decreased beyond 40 dpi. In bacterized plants, Tr was significantly improved at 25°C at 19 dpi but decreased after 26 and 33 dpi regardless of temperature, compared to the non-bacterized plants.

In control plants, the intracellular concentration of CO_2_ (Ci) decreased with the age of the plant (**Figure 1D**) but increased with temperature after 12, 26, and 3 dpi. However, the *Ci* was improved significantly in the presence of bacteria after 26, 33, and 40 dpi at both temperatures.

### PSII Activity

**FIGURE 2:**
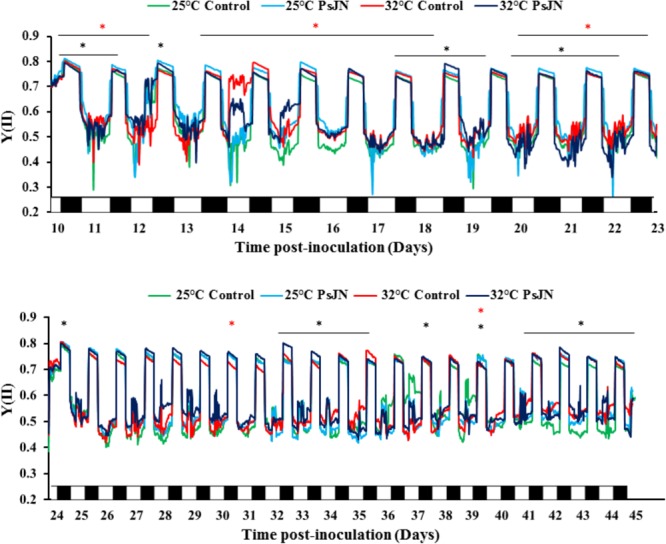
Continuous measurements of fluorescence parameter during treatments with MONITORING-PAM. Real-time efficiency of PSII (YII) during the night (black) and the day (white) throughout the cycle of bacterized and non-bacterized tomato plants at 25 and 32°C. Measurements were monitored every 20 min in chlorophyll fluorescence intensity. Red star (^∗^) and black star (^∗^) represent the significant impact of PsJN at 25 and 32°C, respectively. Data are averages of three independent experimental replicates, each with three plants per treatment (*n* = 9).

The efficiency of the PSII, Y (II), was controlled during the night and the day in both conditions, at 25 and 32°C (**Figure 2**). The results showed that at 25°C, Y (II) of bacterized tomato plants was significantly greater or equal compared to that of the control. At 32°C, the effect of the bacteria was less clear: higher at 10, 14, 17, and 30 dpi, and lower at 12, 18, and 22 dpi.

### Pigment Changes

**FIGURE 3:**
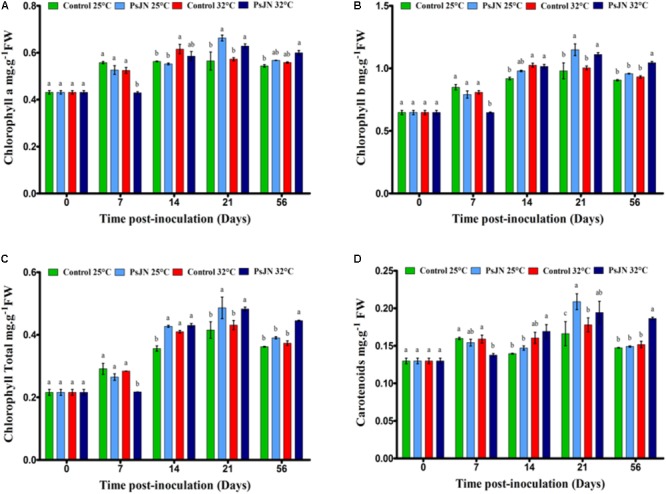
Kinetics of pigments content in tomato leaves of bacterized and non-bacterized plants growing under at 25 or 32°C. Chlorophyll a **(A)**, chlorophyll b **(B)**, total chlorophyll **(C)**, and carotenoid **(D)** concentration. Same letters indicate non-significant differences among all conditions. Data are means ± SE (Standard error) from three independent experimental replicates, each with three plants per treatment (*n* = 9).

At 25°C, the chlorophyll a, b, and carotenoid contents increased through the stages of development (**Figure 3**). At 32°C, the chlorophyll a and b contents increased after 14 dpi and the carotenoids after 21 dpi. The presence of bacteria increased the chlorophyll a, b, and carotenoid contents at 21 dpi for both culture conditions, but caused a decrease at 7 dpi at 32°C. The total chlorophyll content increased in the bacterized plants after 14 and 21 dpi at 25°C and after 21 and 56 dpi at 32°C compared to the control.

### Effect on Carbohydrates

**FIGURE 4:**
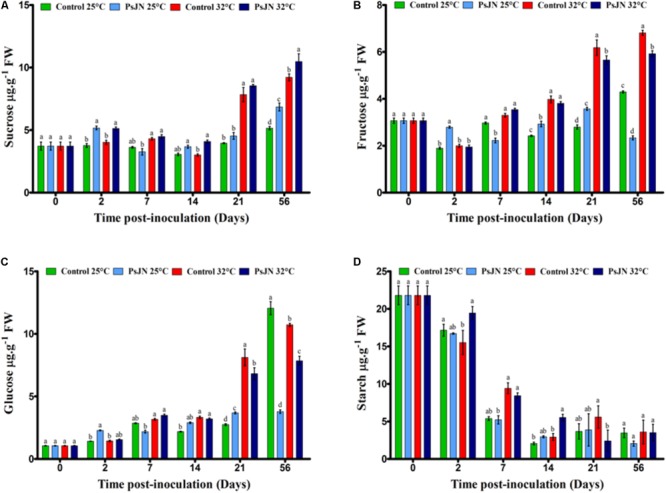
Impact of *P. phytofirmans* strain PsJN on carbohydrates levels. Sucrose **(A)**, fructose **(B)**, glucose **(C)**, and starch **(D)** contents in bacterized and non-bacterized tomato growing under normal (25°C) and high-temperature (32°C) conditions. Data (means ± SE) are averages of three independent experimental replicates, each with three plants per treatment (*n* = 9). Same letters indicate non-significant differences among all conditions.

The effect of PsJN on soluble sugars and the starch accumulation at 25 and 32°C was investigated. The sucrose content increased with the increased temperature mainly after day 21 at 32°C (**Figure 4A**). At 25°C, the presence of bacteria led to a significant increase in the sucrose content at 2 and 56 dpi, while at 32°C, the increase was observed after 2, 14, and 56 dpi.

The level of fructose was significantly higher in plants at 32°C compared to that in plants at 25°C from 14 dpi (**Figure 4B**). At 25°C, the level of fructose in the inoculated tomatoes was higher than in the uninoculated tomatoes after 2, 14, and 21 dpi, while, after 7 and 56 dpi, the level of fructose decreased in the presence of bacteria. At 32°C, the fructose content was low in bacterized plants after 21 and 56 dpi.

The glucose level was significantly higher in the plants at 32°C compared to the plants at 25°C from 14 to 21 dpi (**Figure 4C**). At 25°C, the glucose level in the bacterized tomatoes was higher than in the non-bacterized tomatoes after 2 and 21 dpi, while after 56 dpi, the glucose level decreased in the presence of bacteria. At 32°C, the glucose content was lower in the bacterized plants at 21 and 56 dpi.

In the early stages of tomato development, the starch content was approximately 23 μg/g and decreased during the development stages. Without bacteria, there was no significant difference between the starch content at both temperatures except at 2 dpi, when the starch decreased when the plants were subjected to 32°C. The level of starch was not affected by the presence of PsJN at 25°C but was improved after 2 and 14 dpi when tomato plants were bacterized with PsJN at 32°C (**Figure 4D**).

### Effect on Metabolites

**FIGURE 5:**
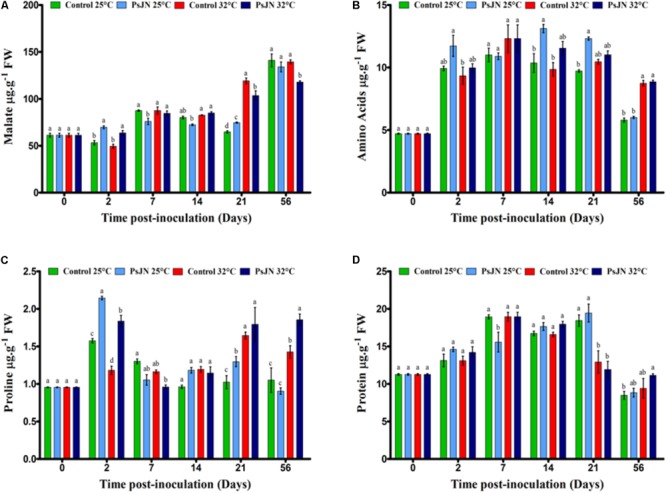
Impact of *P. phytofirmans* strain PsJN on metabolites. Malate **(A)**, amino acids **(B)**, proline **(C)**, and protein **(D)** contents in bacterized and non-bacterized tomatoes under normal (25°C) and high-temperature (32°C) conditions. Data (means ± SE) are averages of three independent experimental replicates, each with three plants per treatment (*n* = 9). Same letters indicate non-significant differences among all conditions.

The concentration of malate increased through the development of the plant, with the highest accumulation measured after 56 days (**Figure 5A**). The increase in temperature increased the malate content in the 21st dpi. At 25°C, the presence of *P. phytofirmans* caused an increase in malate on the second day, followed by a decrease in the 7th dpi before a second increase in the 21st dpi. At 32°C, the bacteria improved malate concentration after 2 dpi but led to a decrease beyond 21 dpi.

The total amino acid level was higher in the bacterized tomatoes after 14 and 21 dpi compared to that in the control plants at 25°C (**Figure 5B**). In addition, tomatoes at 25°C had the same level of amino acids as at 32°C, except after 56 dpi.

Our results revealed that the proline content was significantly higher at 32°C at 21 and 56 dpi compared to that at 25°C (**Figure 5C**). In addition, the presence of bacteria increased the proline content at 32°C after 21 and 56 dpi.

The results showed that the protein content was significantly higher in plants at 25°C than at 32°C at 21 dpi (**Figure 5D**). Based on the findings, in the tomatoes inoculated with PsJN, the protein content was maintained at 25 and 32°C, except at 7 dpi at 25°C.

### Effects on Genes Expression in Tomato Leaves

**FIGURE 6:**
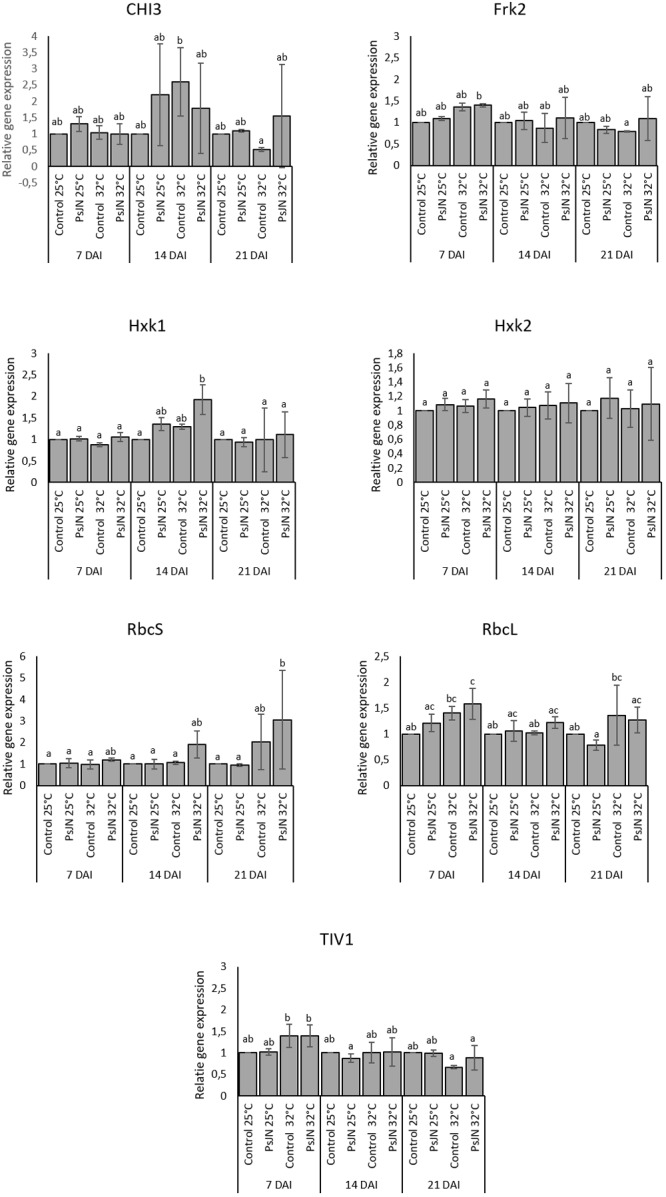
*CHI3, Frk2, Hxk1, Hxk2, RbcS, RbcL*, and *TIV1* in bacterized and non-bacterized tomato under normal (25°C) and high-temperature (32°C) conditions. Same letters indicate non-significant differences among all conditions. Fold expression values are normalized against *EF1a* and *Actin* genes as controls.

The expressions of an acid endochitinase (*CHI3*), an acid invertase (*TIV1*), a fructokinase (*Frk2*), two hexokinases (*Hxk1* and *Hxk2*), ribulose-1,5-bisphosphate carboxylase/small oxygenase (*RbcS*), and large subunits (*RbcL*) at 7, 14, and 21 dpi at 25 and 32°C were monitored. The analysis of gene expressions did not indicate significant changes in their patterns whatever the treatment (**Figure 6**).

**FIGURE 7:**
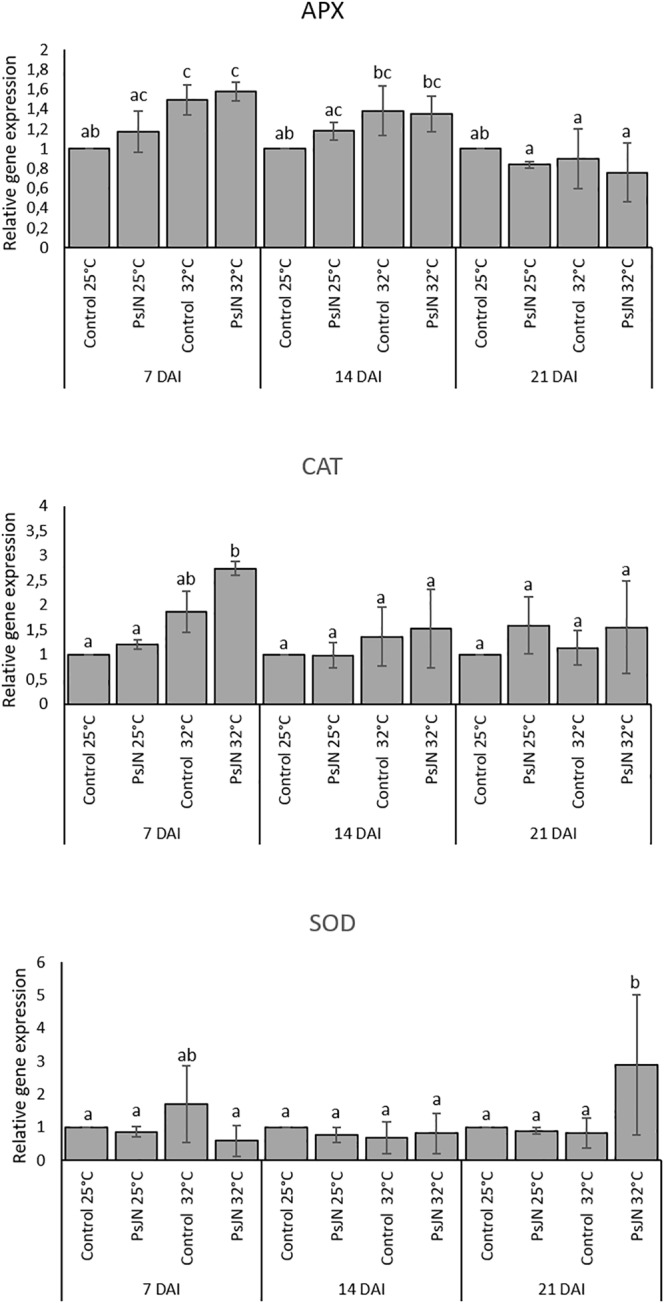
Expression levels of *APX2, CAT1*, and *SOD* in bacterized and non-bacterized tomato under normal (25°C) and high-temperature (32°C) conditions. Same letters indicate non-significant differences among all conditions. Fold expression values are normalized against *EF1a* and *Actin* genes as controls.

In addition, the expression of an ascorbate peroxidase (*APX2*), a catalase (*CAT1*), and a superoxide dismutase (*SOD*) were also analyzed at 7, 14, and 21 dpi at 25 and 32°C. The results indicate that the expression of APX was enhanced at 7 and 14 days after heat stress whatever the treatment. Similarly, the expression of *CAT1* was increased but only at 7 and 14 days after heat stress in both bacterized and non-bacterized plants, but with more impact in bacterized plants. The expression of SOD did not indicate significant changes in its pattern whatever the treatment (**Figure 7**).

## Discussion

Global warming will result in climate change, including higher temperatures, and these changes will cause devastating damage to crop production. In the present study, we investigated the impact of the PsJN strain of *P. phytofirmans* to induce tolerance to high temperatures.

The present study clearly demonstrates the intimate association between *P. phytofirmans* strain PsJN and the tomato plants that grow on non-sterile soil. After the inoculation of the soil, the colonization of the tomato plants by the PsJN strain of *P. phytofirmans* was carried out in different stages. First, the root surface was quickly colonized by bacteria. Similar patterns of rhizoplane colonization have been explained for another plant–bacteria interaction (Compant et al., 2005b; Su et al., 2015). The plants exude the microbial fuel activities in the rhizosphere facilitating the union, which explains the observed population of PsJN after the inoculation of the soil with the bacteria. In the present study, the colonization of the rhizoplane has been controlled by plants that grow in non-sterile soil with the presence of natural microbial communities, which can compete with the inoculated strain to obtain nutrients and inhabit the space (Raaijmakers et al., 2002).

This competition could explain the epiphytic population of the PsJN strain observed 1 week after soil inoculation. However, *P. phytofirmans* survived in the presence of other microorganisms and continued to colonize the root surface, demonstrating its competence in the rhizosphere. After colonizing the root surface, *P. phytofirmans* strain PsJN colonized the interior of the roots and the stem, but only a week later. Probably, the competition between the PsJN strain and other microorganisms in the rhizoplane could have delayed the systemic propagation of the PsJN strain in the interior. *P. phytofirmans* strain PsJN is able to colonize a variety of genetically unrelated plants, such as potato and tomato, corn, switchgrass, Arabidopsis, and grapevine (Conn et al., 1997; Nowak, 1998; Ait Barka et al., 2000; Kim et al., 2012; Su et al., 2015), both endophytically and at the rhizoplane level. In addition, endophytic bacteria could be of particular interest since they have the advantage of being relatively protected from the competitive environment, high stress of the soil, and also from other natural rhizospheric microbiomes (Compant et al., 2005a).

In the present study, *P. phytofirmans* PsJN promoted the growth of tomato plants, confirming previous reports on the PGPR effect of this bacterium (Ait Barka et al., 2000; Naveed et al., 2014a,b).

It is known that during its association with the host, PGPR not only influence growth and yield but also induce plant resistance to abiotic stress. However, the mechanisms behind this interaction are poorly deciphered. The objective of this study was to better understand how the presence of PGPR could influence the photosynthetic mechanisms of tomato plants in response to moderately high-temperature conditions (32°C) and, therefore, contribute to the acclimatization of the plant.

### *Paraburkholderia phytofirmans* Strain PsJN Enhanced the Photosynthesis of Tomato Plants

It has been recognized that photosynthesis is sensitive to environmental stresses and decreases when environmental stress alters any component of photosynthesis. The ability of a plant to maintain the exchange of foliar gasses and the rates of assimilation of CO_2_ under thermal stress is directly related to heat tolerance (Yang et al., 2006). Our results indicate that Pn increases with high temperatures until day 26 and then begins to decrease with the aging of tomato plants. In several plant species, the optimal growth temperature, which maximizes the photosynthetic rate, increases with increasing growth temperature (Hikosaka et al., 2005). Moreover, in eight tomato cultivars that grow under heat stress conditions, the photosynthetic rate was higher in the vegetative stage of the heat-tolerant genotypes compared to the heat-sensitive genotypes; the peak was observed in the flowering stage and the photosynthetic rate decreased in the fruit stage (Abdelmageed and Gruda, 2009), confirming our data. The main cause of reduced Pn could be changed in g_w_ and *Ci* (Sawicki et al., 2017). Furthermore, Sicher (2013) revealed that the net rate of photosynthesis of soybean leaves increased by 20% at 36°C compared to 28°C. In heat-tolerant tomato genotypes exposed to high temperatures, photosynthesis, and transpiration were significantly higher than in non-tolerant genotypes in seedlings, flowering, and early stages of the fruit (Nkansah and Ito, 1994).

After the flowering stage, the Pn of bacterized tomato plants was significantly improved under high-temperature conditions compared to non-bacterized plants. This result indicated that an increase in the rate of assimilation of CO_2_ by the Calvin cycle and the PSII process was due to the presence of the PsJN strain. Haldimann and Feller (2004) showed that photosynthesis and *Ci* increased significantly when the leaf temperature increased to 45°C. The Pn of PGPR host plants increased as a result of improved nutritional status (Giri et al., 2003). In our experiment, the highest level of the net rate of photosynthesis was reached in bacterized tomatoes with PsJN at 32°C compared to 25°C. These results confirmed that *P. phytofirmans* significantly improves photosynthesis, physiology, and growth performance under abiotic stress (drought) (Naveed et al., 2014b).

Stomatal closure is a crucial factor in regulating the net photosynthetic rate and is, therefore, a key factor in regulating the global carbon cycle and plant carbon metabolism. In addition, stomatal closure (g_w_ reduction) reduces the availability of carbon dioxide, making plants more susceptible to photodamage (Lawlor and Cornic, 2002).

Stomatal conductance (stomatal opening) in bacterized tomato plants was significantly higher (*P* < 0.0001) than in non-bacterized tomato plants and increased with the rate of temperature increase. Similarly, Cheng et al. (2009) found that g_w_ and *Ci* were significantly higher in tomato plants than in wild-type tomatoes under heat stress. Moreover, Rizhsky et al. (2004) reported that the opening of stomata is improved under heat stress. As a consequence, stomatal regulation is one of the key factors that regulate local growth.

### Effect of *Paraburkholderia phytofirmans* Strain PsJN on Chlorophyll Fluorescence

The protein complex of the PSII is the most vulnerable component of the photosynthetic machinery in response to abiotic stress. The inactivation of PSII due to thermal stress is related to damage on the donor side, the reaction center, and the acceptor side of the photosystem’s electronic transport chain (Wise et al., 2004). Our results reveal that the presence of PsJN bacteria induced the efficiency of PSII compared to non-bacterized plants, with the greatest intensity when the plants were exposed to 32°C. These results suggest that the net photosynthetic rate as well as the maximum quantum efficiency of the PSII improved, reflecting the increase in CO_2_ assimilation in the presence of strain PsJN. The damage to the photosynthetic efficiency was measured as a change in the Fv/Fm ratio. A significant decrease in Fv/Fm suggested an increase in the energy dissipated as heat and photoinhibition for the photosynthetic apparatus. Sharma et al. (2015) found that the maximum potential quantum efficiency of the PSII ratio (Fv/Fm) was significantly correlated with g_w_ and Pn in wheat cultivars at high temperature 36/30°C. Furthermore, the efficiency of PSII was significantly increased in *Pinus halepensis* inoculated with *Pseudomonas fluorescens* Aur6 (Rincón et al., 2008). These observations indicate that the PsJN strain of *P. phytofirmans* has a positive effect on the performance efficiency of the PSII.

The inhibition of PSII causes a change in the variable fluorescence of chlorophyll a, and chlorophyll *in vivo* can be used to detect the changes in the photosynthetic apparatus that reflects the performance of photosynthesis. As a non-intrusive method, chlorophyll fluorescence analysis can detect the effects of environmental stresses on plants. In addition, it provides information on the capacity of a plant to tolerate environmental stresses. According to the recorded data, the high temperature (32°C) induced the modification of the quantum yield of PSII in non-bacterized tomato plants compared to a similar treatment at 25°C. The rapid rise in fluorescence intensity is the result of a decrease in electron acceptors or donors beyond PSII and is subject to high-temperature processing.

### Effect of *Paraburkholderia phytofirmans* Strain PsJN on the Pigment Contents

Increases in pigment contents, including chlorophyll a, chlorophyll b, total chlorophyll, and carotenoid contents, at high temperature may be associated with the photosynthetic elevation performance. Our study suggests that the tomato plants were well acclimated, implying that the Tom Red in drum belongs to heat-tolerant cultivars. Similarly, several reports have shown that the chlorophyll content increased, particularly at high temperatures in a heat-tolerant tomato cultivar (Camejo et al., 2005; Zhou et al., 2015; Nankishore and Farrell, 2016). These results confirm the hypothesis that the ability of plants to increase and/or maintain the chlorophyll content at high temperatures is crucial for the thermal tolerance of tomato genotypes.

Our results found that the presence of *P. phytofirmans* strain PsJN improved the chlorophyll leaf content in tomato plants under normal and high-temperature conditions. This further confirms the benefits of the PsJN strain of *P. phytofirmans* to promote the fitness of tomato plants and their ability to confer tolerance to high temperatures. According to our results, the chlorophyll content of the wheat flag leaf inoculated with PsJN increased compared to PsJN under drought stress (Naveed et al., 2014a). Similarly, the pigment content and the photosynthetic efficiency (chlorophylls and carotenoids) were improved in sorghum seedlings inoculated with the AKM-P6 strain of *Pseudomonas* sp. at high temperatures (Ali et al., 2009). In addition, Zhang et al. (2008) found that *Bacillus subtilis* GB03 improves the photosynthetic efficiency and the chlorophyll content of *A. thaliana* through the modulation of endogenous glucose signaling and the detection of abscisic acid.

### Effect of the Bacterium *Paraburkholderia phytofirmans* Strain PsJN on the Biochemical Status Within Tomato Leaves

The accumulation of osmolytes such as proline and sugars is a well-known adaptation mechanism in plants against abiotic stress conditions, including high temperature, since heat-sensitive plants apparently lack the capacity to accumulate these substances. The present study showed that the metabolism in bacterized and non-bacterized tomato plants depended on temperature.

#### Soluble Sugars and Starch

The parameters of plant growth, such as cell division, expansion, differentiation, and maintenance require carbon and energy. Carbohydrates are produced in the leaves of plants (source) by photosynthetic activity and transported to the sink parts (flowers, fruits, seeds, and root system). Sugars play a vital role in maintaining the growth of source and sink tissues, as well as signaling molecules under abiotic and biotic stress (Roitsch, 1999; Eveland and Jackson, 2011).

Our findings revealed that soluble sugars (glucose, fructose, and sucrose) and starch levels in tomato leaves were significantly affected by high temperature. Similarly, the level of sucrose quadrupled in tomato leaves under heat stress at 35°C compared to the control at 25°C (Rivero et al., 2014), while glucose and fructose levels decreased compared to that in plants not subjected to stress at 25°C (Jie et al., 2012). Interestingly, sucrose can also be replaced by proline in the plants as the main osmoprotectant under heat stress (Rizhsky et al., 2004). The results indicated that sugars were accumulated in PsJN-bacterized tomato plants in both conditions (25 and 32°C). The highest level of sucrose observed in bacterized tomato plants was at 32°C, 56 dpi. In addition, the fructose and glucose contents were negatively impacted at 56 dpi for both conditions after 21 dpi.

The highest starch content was observed in the vegetative stage of the tomato plants (19 days), while it was lower in the fully established fruit where there was a limitation of the sink (40 days) (Li et al., 2015). Similarly, our finding showed the highest accumulation of starch in the tomato leaves in the early growth period (20 days, sink limitation) and tended to be lower in full fruiting. The lowest level of starch at 21 days after planting could be due to the period of sink strength in the tomato plants during flowering and the initial stages of fruit set. The starch content in tomato leaves was significantly affected by bacterial inoculation at 32°C after 7 dpi. The increase in carbohydrates, specifically starch, correlated with grapevine tolerance to biotic and abiotic stress (Ait Barka et al., 2006; Miotto-Vilanova et al., 2016).

#### Organic Acids (Malate)

Malate may accumulate in the vacuole or act as a source of NADH in the cytosol and drives increased mitochondrial production of ATP (Scheibe, 2004). From our findings, malate increased linearly with the growth of tomato plants (bacterized and non-bacterized) under both temperature conditions. Similar results were reported in soybean leaflets exposed to heat 36/28°C in which malate increased compared to leaflets exposed to 28/20°C (Sicher, 2015). In addition, in *A. thaliana*, an increase in the malate content was observed in plants exposed to high temperatures (Kaplan et al., 2004). Our findings suggest that malate may coordinate the promotion of heat tolerance at 32°C by affecting the release of energy (ATP content) associated with the photosynthetic rates (mentioned earlier in the gas exchange section) in non-bacterized and bacterized tomato plants.

#### Total Amino Acids and Proline

The total amino acids of bacterized tomato plants reached the highest level during the flowering phase and the first stage of fruit. Similar results were reported in perennial ryegrass treated with exogenous amino acids (Botta, 2012). In addition, in *A. thaliana* plants exposed to heat stress (40°C), amino acids, fructose, and sucrose with 58 metabolites (including malate) increased during the first half hour of heat stress (Kaplan et al., 2004). Ali et al. (2011) revealed an increase in the amino acid and proline contents in wheat inoculated with the AKMP7 strain of *Pseudomonas putida* under heat stress. As amino acids, proline supports cellular proteins and acclimatization of membranes to extreme temperatures and other abiotic stresses such as salt and cooling (Ait Barka et al., 2006; Chen et al., 2007; Grover et al., 2011).

#### Proteins

With the exception of the 21st dpi, the protein content was stable at high temperature, probably due to an accumulation of compatible solutes such as sucrose, amino acids, and malate, which are able to stabilize proteins when the acquired thermotolerance occurs. Our results suggest that the high level of sucrose can act as a signaling molecule that prevent the degradation of proteins under heat stress. Similarly, Arbona et al. (2013) reported that simple sugars play a crucial role as compatible solutes that support the cell volume, the thermostability of cell membranes, and the inhibition of protein damage. Our results are supported by Guo et al. (2014) finding that a more pronounced increase in protein synthesis occurs during the flowering period than in the vegetative phase in pepper plants subjected to high temperatures (40°C). We could assume that the protein level decreased in the leaves of the bacterized and non-bacterized tomato plants to meet the requirements for flower production under abnormal conditions at 32°C during the flowering stage.

### Gene Expression in Tomato Leaves

Previous studies on tomato genes were carried out to study plant responses to different stresses (Subramanian et al., 2015; Zhang et al., 2018). Our study revealed that there are no significant changes in the expression of genes related to the photosynthesis in tomato leaves either in bacterized or non-bacterized plants. The expression of photosynthesis-related genes as well as that of defense-related genes could be more probably regulated at the post-transcriptional level. Similarly, the leaves of the *A. thaliana* plants inoculated with the PsJN strain of *P. phytofirmans* did not significantly alter the expression of the defense genes (PR1 and PDF1.2) in response to cold stress (Su et al., 2017). However, the expression of genes related to the reactive oxygen species (ROS) reveals a higher induction of CAT1 and APX2 under high temperature in the leaves of tomato plants. In accordance, several reports indicate that a higher expression of CATs and APXs under high temperature may protect plants from the ROS produced after exposure to a high temperature since it has been suggested that under different kinds of stresses, the acquisition of tolerance is closely related with ROS removal (Møller, 2001; Møller et al., 2007).

## Conclusion

**FIGURE 8:**
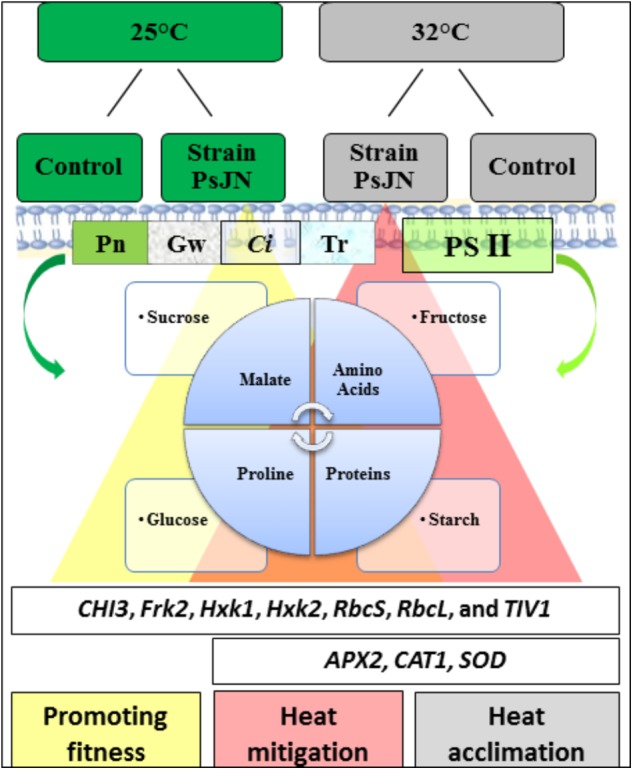
A schematic model on the impact of strain PsJN on tomato growth in both the sugar metabolites and compatible solutes as primary signaling, which play a pivotal role in alleviating the deleterious of high temperature. Increasing a level of compounds in the presence of PsJN (yellow and red triangle combined with its results (same colors).

The response of the tomato plants indicated an improvement of the photosynthetic features that show that the PSJN strain of *P. phytofirmans* plays a key role in promoting tomato growth at high temperature. Sugars, total amino acids, proline, and malate also accumulated significantly, playing a fundamental role in improving heat tolerance and preventing the degradation of starch in bacterized tomato plants. Consequently, the physiological changes in the rate of photosynthesis and fluorescence of chlorophyll, and other biochemical parameters indicate that the cultivar Tom Red was well acclimatized. The study showed that the presence of *P. phytofirmans* strain PsJN might mitigate the adverse effects of high temperatures by increasing plant growth and promoting gas exchange. The use of this bacterium alone can alleviate heat stress in agriculture, opening up new and emerging microbial applications. **Figure 8** summarizes a hypothetical model, explaining the potential mechanisms relating to the inoculation of PsJN facilitates the alleviation of heat stress at physiological and molecular levels.

## Author Contributions

AI, NV-G, and EAB designed the research. AI, QE, LS, BC, YG, PB, NV-G, and EAB carried out the experiments and analysis and interpretation of data. AI, QE, LS, YG, PB, CC, CJ, NVG, and EAB wrote the manuscript with contributions and discussion from all of the coauthors. All authors have given approval to the final version of the manuscript.

## Conflict of Interest Statement

The authors declare that the research was conducted in the absence of any commercial or financial relationships that could be construed as a potential conflict of interest.

## Footnotes

1 http://www.ncbi.nlm.nih.gov/tools/primer-blast

## Supplementary Material

FIGURE S1 Impact of the *P. phytofirmans* strain PsJN on tomato fresh weight after soil inoculation. Values shown are means ± SD of three independent repetitions (each repetition was realized in triplicates). Data (means ± SE) are averages of three independent experimental replicates, each with three plants per treatment (*n* = 9). Same letters indicate non-significant differences among all conditions.

TABLE S1 List of specific primers used in qPCR (designed in this study).

TABLE S2 Epiphytic and endophytic colonization of tomato by *P. phytofirmans* strain PsJN after soil inoculation with 5 × 10^7^ CFU of PsJN::*gfp*. Data (log_10_ CFU/g FW) ± SD represent means of three independent experimental replicates, each with three plants per treatment (*n* = 9). FW: fresh weight, ND: not detectable.

## Abbreviations

*Ci*: intercellular CO_2_ concentration
Tr: transpiration rate
Fv/Fm: maximum efficiency of PSII
g_w_: stomatal conductance
*P. phytofirmans* PsJN: *Paraburkholderia phytofirmans* strain PsJN
PGPR: plant growth-promoting rhizobacteria
Pn: net photosynthesis
PSII: photosystem II
RbcL: RuBisCO large subunit
RbcS: RuBisCO small subunit
RuBisCO: ribulose-1,5-bisphosphate carboxylase/oxygenase
PSII: effective quantum yield of PSII
