# Supplementary figures and images for: Impacts of Paraburkholderia phytofirmans Strain PsJN on Tomato (Lycopersicon esculentum L.) Under High Temperature
Source: Front Plant Sci. 2018 Oct 18;9:1397. doi: 10.3389/fpls.2018.01397 (PMC6201190; doi:10.3389/fpls.2018.01397)

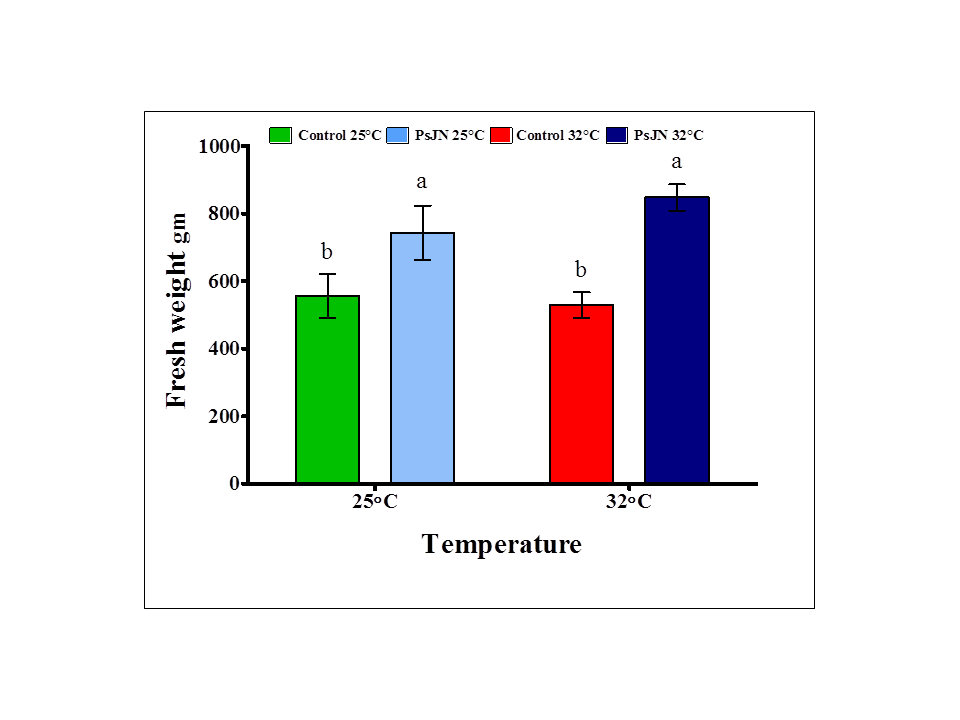

Supplement: FIGURE S1 — Impact of the P. phytofirmans strain PsJN on tomato fresh weight after soil inoculation. Values shown are means ± SD of three independent repetitions (each repetition was realized in triplicates). Data (means ± SE) are averages of three independent experimental replicates, each with three plants per treatment (n = 9). Same letters indicate non-significant differences among all conditions. [file Image_1.TIFF]
