# Supplementary material for: Impacts of Paraburkholderia phytofirmans Strain PsJN on Tomato (Lycopersicon esculentum L.) Under High Temperature
Source: Front Plant Sci. 2018 Oct 18;9:1397. doi: 10.3389/fpls.2018.01397 (PMC6201190; doi:10.3389/fpls.2018.01397)
Supplement: TABLE S2 — Epiphytic and endophytic colonization of tomato by P. phytofirmans strain PsJN after soil inoculation with 5 × 10^7^ CFU of PsJN::gfp. Data (log10 CFU/g FW) ± SD represent means of three independent experimental replicates, each with three plants per treatment (n = 9). FW: fresh weight, ND: not detectable. [file Data_Sheet_1.PDF]

Days post inoculation	Rhizoplane		Endorhiza		Stem		Leaves	
	25°C	32°C	25°C	32°C	25°C	32°C	25°C	32°C
**0**	6.85 ± 0.04	6.86 ± 0.03	ND		ND		ND	
**2**	6.07 ± 0.05	6.03 ± 017	ND		ND		ND	
**7**	5.98 ± 0.29	5.45 ± 0.31	4.05 ± 0.19	4.09 ± 0.18	3.31 ± 0.40	3.77 ± 0.69	ND	
**14**	5.22 ± 0.22	4.9 ± 0.24	3.88 ± 0.04	4.11 ± 0.26	2.25 ± 0.53	2.09 ± 0.14	ND	
**21**	4.8 ± 0.39	4.82 ± 0.20	2.98 ± 0.21	3.30 ± 0.54	1.61 ± 0.56	1.35 ± 0.14	ND	

Table S2. Epiphytic and endophytic colonization of tomato by *P. phytofirmans* strain PsJN after soil inoculation with 5 x 10^7 CFU of PsJN::*gfp*. Data ($\log_{10}$ CFU/g FW) ± SD represent means of three independent experimental replicates, each with three plants per treatment (n = 9).. FW: fresh weight, ND: not detectable.
